# In regard to P. von Platen et al., “The dawn of physiological closed-loop ventilation—a review”

**DOI:** 10.1186/s13054-020-03042-x

**Published:** 2020-06-10

**Authors:** Fleur T. Tehrani

**Affiliations:** School of Engineering and Computer Science, California State University, Fullerton, California USA

This letter is regarding a recent review article in *Critical Care* [1].

Platen et al. [1] refer to another review article but do not cite any of the references of that article by Tehrani that describe a system known as adaptive support ventilation (ASV) except a conference paper in 1991. No other articles that describe the same main features of a system marketed as IntelliVent-ASV are cited either. Tables 1 and 2 of the paper by Platen et al. [1] provide no reference to any of the publications by Tehrani et al. on either control of ventilation or control of oxygenation.

Even when Platen et al. [1] refer to a 1991 conference paper by Tehrani, they make misleading and incorrect statements. They claim the idea of minimization of the respiratory work, as proposed by Otis et al. [2] was used by Mitamura et al. [3]. The hypothesis by Otis et al. [2] that breathing frequency is optimized to minimize the respiratory work rate was not in relation to positive-pressure mechanical ventilation, which was not even used in practice in 1950. That hypothesis was confirmed by some and challenged by some physiologists until it was used for the first time, along with modifications, and many other features in an invention to automatically control mechanical ventilation in synchrony with a patient’s natural breathing. A patent covering that invention was issued in 1991 [4]. One of the embodiments of that patent is known as ASV, and the product has been marketed under license of that patent. The details of how the manufacturer of ASV used the invention and eventually had to get a license on the patent have already been published and are not repeated here for brevity. Despite what is claimed in the paper by Platen et al. [1], Mitamura et al. [3] rejected the approach by Otis et al. [2] for not producing realistic results and adopted the work of Mikami and Yoshimoto [5] (see Figure 4 in [3]).

In brief, an objective review should not ignore or undermine some of the important contributions to a field.

## Authors’ Response

Philip von Platen, Anake Pomprapa, Burkhard Lachmann, Steffen Leonhardt

The great contribution by Dr. Tehrani and her team to the field of closed-loop control of ventilation is unquestionable, as clearly shown by the high citations of their work. However, given the restricted number of allowed references, the aim of our review was not to strive for completeness, but instead show the evolution of physiological closed-loop control (PCLC) on its way to clinical evidence. As such, we only considered literature with closed-loop evaluations in large animals or patient studies, as explicitly mentioned in the paper. Many of the important works by Dr. Tehrani do not fall within this scope, such as the computerized decision support system evaluated only in the open loop [6].

To highlight the early and important contribution by Dr. Tehrani, the conference paper, which is the earliest publication excluding the patent, has been cited by us in conjunction with adaptive support ventilation (ASV). The reader is made aware of the parallels between these concepts without diverging into the legal dispute about Dr. Tehrani’s invention. The extension of Dr. Tehrani’s own work was evaluated in simulations and an animal trial with six pigs [7]. ASV has become commercially available and has therefore been clinically evaluated. This clinical evaluation is the final test for PCLC systems and a requirement for the acceptance from clinicians. Of course, ASV has been covered in our review.

We would further like to clarify the statement about Mitamura et al.’s [3] work being “closely related” to the concept of Otis et al. [2], as stated in the manuscript. Mitamura et al. mentioned in their abstract: “… respiratory rate is computed to minimize ventilatory work” [3]. They subsequently acknowledged the work by Otis et al. [2] but found that for higher levels of alveolar ventilation, the work by Mikami and Yoshimoto approximated the data better [3]. We agree that the work by Mitamura et al. can be considered as a modification of Otis et al.’s work, but the core concept remains similar. This relationship has also been acknowledged elsewhere [8].

In conclusion, we did not “ignore or undermine some of the important contributions to the field”. Dr. Tehrani’s publications are important contributions to the field, but several of her papers did not meet our mentioned paper selection criteria. In addition, a review on a topic as vast as physiological closed-loop control of mechanical ventilation must, unfortunately, exclude some literature to retain a defined scope and adhere to the limited number of allowed references.

We sincerely hope that this response clarifies the situation.

## Data Availability

Not applicable
